# Effects of *Escherichia Coli* Subtilase Cytotoxin and Shiga Toxin 2 on Primary Cultures of Human Renal Tubular Epithelial Cells

**DOI:** 10.1371/journal.pone.0087022

**Published:** 2014-01-21

**Authors:** Laura B. Márquez, Natalia Velázquez, Horacio A. Repetto, Adrienne W. Paton, James C. Paton, Cristina Ibarra, Claudia Silberstein

**Affiliations:** 1 Laboratorio de Investigaciones Biomédicas, Departamento de Fisiología, Instituto de Fisiología y Biofísica Bernardo Houssay (IFIBIO Houssay-CONICET), Facultad de Medicina, Universidad de Buenos Aires, Paraguay, Buenos Aires, Argentina; 2 Departamento de Pediatría, Facultad de Medicina, Universidad de Buenos Aires, Buenos Aires, Argentina; 3 Research Centre for Infectious Diseases, School of Molecular and Biomedical Science, University of Adelaide, Adelaide, S.A., Australia; 4 Laboratorio de Fisiopatogenia, Departamento de Fisiología, Instituto de Fisiología y Biofísica Bernardo Houssay (IFIBIO Houssay-CONICET), Facultad de Medicina, Universidad de Buenos Aires, Buenos Aires, Argentina; Institut Curie, France

## Abstract

Shiga toxin (Stx)-producing *Escherichia coli* (STEC) cause post-diarrhea Hemolytic Uremic Syndrome (HUS), which is the most common cause of acute renal failure in children in many parts of the world. Several non-O157 STEC strains also produce Subtilase cytotoxin (SubAB) that may contribute to HUS pathogenesis. The aim of the present work was to examine the cytotoxic effects of SubAB on primary cultures of human cortical renal tubular epithelial cells (HRTEC) and compare its effects with those produced by Shiga toxin type 2 (Stx2), in order to evaluate their contribution to renal injury in HUS. For this purpose, cell viability, proliferation rate, and apoptosis were assayed on HRTEC incubated with SubAB and/or Stx2 toxins. SubAB significantly reduced cell viability and cell proliferation rate, as well as stimulating cell apoptosis in HRTEC cultures in a time dependent manner. However, HRTEC cultures were significantly more sensitive to the cytotoxic effects of Stx2 than those produced by SubAB. No synergism was observed when HRTEC were co-incubated with both SubAB and Stx2. When HRTEC were incubated with the inactive SubA_A272_B toxin, results were similar to those in untreated control cells. Similar stimulation of apoptosis was observed in Vero cells incubated with SubAB or/and Stx2, compared to HRTEC. In conclusion, primary cultures of HRTEC are significantly sensitive to the cytotoxic effects of SubAB, although, in a lesser extent compared to Stx2.

## Introduction

Shiga toxin (Stx)-producing *Escherichia coli* (STEC) colonizes the distal small intestine and colon causing watery diarrhea, hemorrhagic colitis, and hemolytic uremic syndrome (HUS) [1], [2]. HUS is the most common cause of acute renal failure in children in many parts of the world and the second leading cause of chronic renal failure in children younger than 5 years [2], [3]. Renal damages have been strongly associated with Shiga toxin type 1 and/or 2 (Stx1, Stx2) [4] produced by *Escherichia coli* O157∶H7 and other related strains frequently isolated from children with HUS, although strains expressing Stx2 are highly prevalent in Argentina [5].

While the production of Stx by STEC is the primary virulence factor responsible for HUS, it was reported that some STEC non-O157 strains produce an additional toxin termed subtilase cytotoxin (SubAB) which may play a role in the pathogenesis of HUS [6], [7]. SubAB was identified for the first time in a virulent O113∶H21 STEC strain that caused an outbreak of HUS in South Australia [8]. The presence of *subAB* genes was further detected in other STEC strains belonging to different serotypes, and in other countries [7], [9]. Recently, it was reported [9] the detection of *subAB* gene in 36% of the cattle strains, and in 32% of human strains of STEC strains isolated in Argentina.

Stx and SubAB cytotoxins are members of two different AB5 toxin families, which contain an A subunit monomer, of 32 kDa and 35 kDa respectively, bound non-covalently to a pentamer of 7.7-kDa and 13-kDa B subunits respectively [6], [10]. However, both toxins bind to different membrane receptors and exert their cytotoxic activity through different cell pathways.

The Stx B subunit pentamer binds to the glycolipid globotriaosylceramide (Gb3) on the plasma membrane of target cells [11], followed by holotoxin internalization into the cell and transport to the endoplasmic reticulum by a retrograde pathway [12]. Stx A-subunit is cleaved by a furin-like protease, releasing the enzymatically active A1-subunit, which is translocated into the cytoplasm where it exhibits RNA *N*-glycohydrolase activity and cleaves a specific adenine residue on the 28S ribosomal RNA in the cytosol, thereby inhibiting protein synthesis [13], [14]. The binding of Stx to renal tubular epithelial cells expressing Gb3 *in vitro* [15], [16] and *in vivo* [15], [17]–[19] has been shown to decrease cell viability, inhibit protein synthesis and induce apoptosis and necrosis. We have previously observed that C-9 (Genzyme, Waltham, MA), a specific inhibitor of glucosylceramide (GL1) synthase, decreases Gb3 expression levels and prevents the cytotoxic effects of Stx2 on primary cultures of human renal tubular epithelial cells (HRTEC) (20). In Sprague-Dawley rats intraperitoneally injected with a filtered bacterial supernatant containing Stx2, oral treatment with C-9 significantly decreased mortality to 50% and reduced the extent of renal and intestinal injuries in the surviving animals [19].

The B subunit pentamer of SubAB binds to the surface of target cells via glycans displayed on glycoproteins [21] that terminate in α2-3-linked *N*-glycolylneuraminic acid (Neu5Gc) [22]. The A subunit, is a subtilase-like serine protease that selectively cleaves the endoplasmic reticulum (ER) chaperone glucose-regulated protein 78 (GRP78, also known as BiP) [23], triggering ER stress signaling pathways and the unfolded protein response (UPR) [24]. This leads to transient inhibition of protein synthesis and cell cycle arrest at G1 phase, and induces caspase-dependent apoptosis via mitochondrial membrane damage in Vero cells and HeLa cells [24]–[27]. Serine protease activity is fundamental to the cytotoxic mechanism of SubAB. Mutation of Ser272→Ala in the A subunit of SubAB completely eliminated serine protease activity and cell cytotoxicity [6]. Studies *in vivo* showed that SubAB caused HUS-like pathologies, which are associated with induction of apoptosis in the liver, kidney and spleen [6], [28].

The purpose of the present work was to study the cytotoxic effects of SubAB on primary cultures of human cortical renal tubular epithelial cells (HRTEC). SubAB studies were performed in parallel with those of Stx2 in order to evaluate and compare their contribution on the renal tubular injury in HUS.

## Materials and Methods

### Reagents

Toxins: Stx2 was purchased at Phoenix Laboratory, Tufts Medical Center, Boston, MA, USA and it was checked for lipopolysaccharide (LPS) contamination by Limulus amoebocyte lysate assay. Stx2 contained <10 pg LPS/ng of pure Stx2. The SubAB and the inactive mutant SubA_A272_B were purified as described previously [6], [23].

### Cell culture

HRTEC primary cultures were isolated from kidneys removed from pediatric patients undergoing nephrectomies, at the “Servicio de Pediatría, Hospital Nacional Prof. A. Posadas”, Buenos Aires, Argentina. Written informed consent from the next of kin, or guardians on the behalf of the children was obtained for use of these samples for research. The Ethics Committee of the Hospital Nacional Prof. A. Posadas approved the use of human renal tissues for research purposes. The cortex was dissected from the renal medulla and the primary cultures were performed according to the methods described previously [29]. The cortical fragments were incubated for 30 min at 37°C in Hank's solution containing 0.1% collagenase type I. Then, the preparation was washed and filtered through a 70 µm pore size cell strainer (BD Bioscience, MA, USA), to discard the glomeruli. The filtered tubules were incubated in Hank's solution containing 0.2% collagenase type 1 (Sigma-Aldrich, St Louis, MO, USA), for 30 min at 37°C. The obtained cells were washed and resuspended in RPMI 1640 medium (HyClone) supplemented with 5% fetal bovine serum (FBS), 2 mM L-glutamine and 100 U/ml penicillin/streptomycin (all from GIBCO BRL, Grand Island, NY, USA). Cells were incubated in 5% CO_2_ atmosphere at 37°C and grown in flasks to confluence. Cells were cultured in flasks in RPMI medium with supplements and 1% endothelial cell growth supplement (Sigma), and used between 3–5 passages. By light microscopy, more than 95% of the cells had similar morphologies. These cells were confirmed as epithelial cells by positive staining for cytokeratins. These cells were also positive stained for aquaporin 1 (Anti-AQP1, Alpha Diagnostic, USA), confirming their origin as proximal tubule epithelial cells. Furthermore, the cells were also negative for the endothelial cell antigen CD31. Depending on the particular experiment, HRTEC cells were grown in 96-well plates, or in glass cover slips in 24-well plates.

For some experiments, the Vero cell line was also cultured in flasks in RPMI 1640 medium supplemented with 10% FBS, 2 mM L-glutamine and 100 U/ml penicillin/streptomycin.

### Neutral red cytotoxicity assay

The neutral red cytotoxicity assay was performed according to the method described previously [29]. HRTEC cells were plated in 96-well plates and grown to sub-confluence in complete RPMI medium. Cells were then washed and exposed to different dilutions of SubAB, SubA_A272_B, or Stx2 under growth-arrested conditions (endothelial growth supplement and serum free-medium) for 24 and 72 h. Two hundred microliters of freshly diluted neutral red in RPMI medium were then added to a final concentration of 50 µg/ml and cells were incubated for an additional 3 h at 37°C in 5% CO_2_. Cells were then washed with 1% CaCl_2_ and 4% formaldehyde, and solubilized in 1% acetic acid and 50% ethanol. Absorption in each well was read in an automated plate spectrophotometer at 546 nm. Results are expressed as neutral red uptake percent, with 100% representing cells incubated under identical conditions but without toxin treatment.

### Apoptosis

*Acridine orange* (AO) - *ethidium bromide* (EB): Apoptosis was analyzed on HRTEC and Vero cells cultured on cover slips for 2 days, immersed in 24-well plates with RPMI medium with supplements. Cells were incubated for 1 h–24 h with 10 ng/ml of SubAB and/or 10 ng/ml of Stx2, in growth arrest conditions. For some experiments cells were incubated with the mutated toxin SubA_A272_B (10 ng/ml). After each treatment, the percentage of apoptotic cells was established morphologically by fluorescence microscopy after staining with acridine orange/ethidium bromide (1∶1, v/v) in a final concentration of 100 µg/ml [30]. Each experiment was performed in duplicate, counting a minimum of 200 total cells per duplicate. The fluorescence was observed with a Nikon model Eclipse E-2000 fluorescence microscope. Images were captured with a digital camera (Nikon E4300) and processed using the Adobe Photoshop 6.0 image analysis software package (Media Cybernetics). Apoptotic cells were defined on the basis of nuclear morphologic changes such as chromatin condensation and staining [30]. Normal cells are permeable to AO but impermeable to EB, while apoptotic and necrotic cells become permeable also to EB. Therefore, live cells show normal coloration of the nuclei, with green chromatin and organized structures; apoptotic cells present chromatin condensation and fragmentation, and a green-yellow or orange coloration, and necrotic cells have similar normal nuclei staining as live cells except the chromatin is red-orange instead of green.

*Annexin V-FITC – Propidium Iodide (AV-PI)*: Apoptosis was also analyzed by AV-PI staining, using the apoptosis detection kit supplied by Sigma-Aldrich. HRTEC cells were cultured on cover slips for 2 days, immersed in 24-well plates with RPMI medium with supplements, as described above. Cells were incubated with SubAB and/or Stx2 for 24 h. Cells were then incubated with a solution of annexin V-FITC and propidium iodide (1∶1 v/v; 2 ug/ml) for 10 min at room temperature. Annexin V-FITC and propidium iodide were detected as green and red fluorescence, respectively, under a fluorescence microscope, as was described elsewhere [31]. Constant optical threshold and filter combination were used. To calculate the percentage of apoptotic and necrotic cells, each experiment was done in duplicate and a minimum of 200 total cells were evaluated per duplicate. Live cells showed no staining by either PI or AV. Cells which were early in the apoptotic process stained with the AV alone. Late apoptotic cells were stained by both AV and PI, while necrotic cells were stained by PI alone.

### Cell proliferation

Cell proliferation rate was measured at different times by incorporation of bromodeoxyUridine (5-Bromo-2-DeoxyUridine, BrdU) into the DNA of cells in S-phase of the cell cycle, and detected using a specific antibody. HRTEC cultures grown on cover slips were incubated with 10 ng/ml of SubAB, SubA_A272_B, or Stx2, for 2 to 24 h. After treatments, cells were pulse-labeled with 10 µM BrdU (Sigma-Aldrich, St Louis, MO, USA) for 2 h at 37°C. Cells were then fixed with 70% ethanol, denatured with 2N HCl and 0.5% triton X-100, and neutralized with 0.1 M Na_2_B_4_O_7_, pH: 8.5. For BrdU detection, indirect immunofluorescence was performed using an antibody against BrdU (Sigma-Aldrich) diluted 1∶100 in PBS with 5% FBS, and 0.05% Tween 20. Alexa fluor 488 goat anti-mouse IgG (Invitrogen) diluted 1∶200 in PBS was used as secondary antibody.

### Statistical analysis

Results are reported as means ± standard error of the mean (SEM). The significance of any differences was determined using the Student's t-test. *P* values <0.05 were considered statistically significant.

## Results

### Cell viability

**Figure 1:**
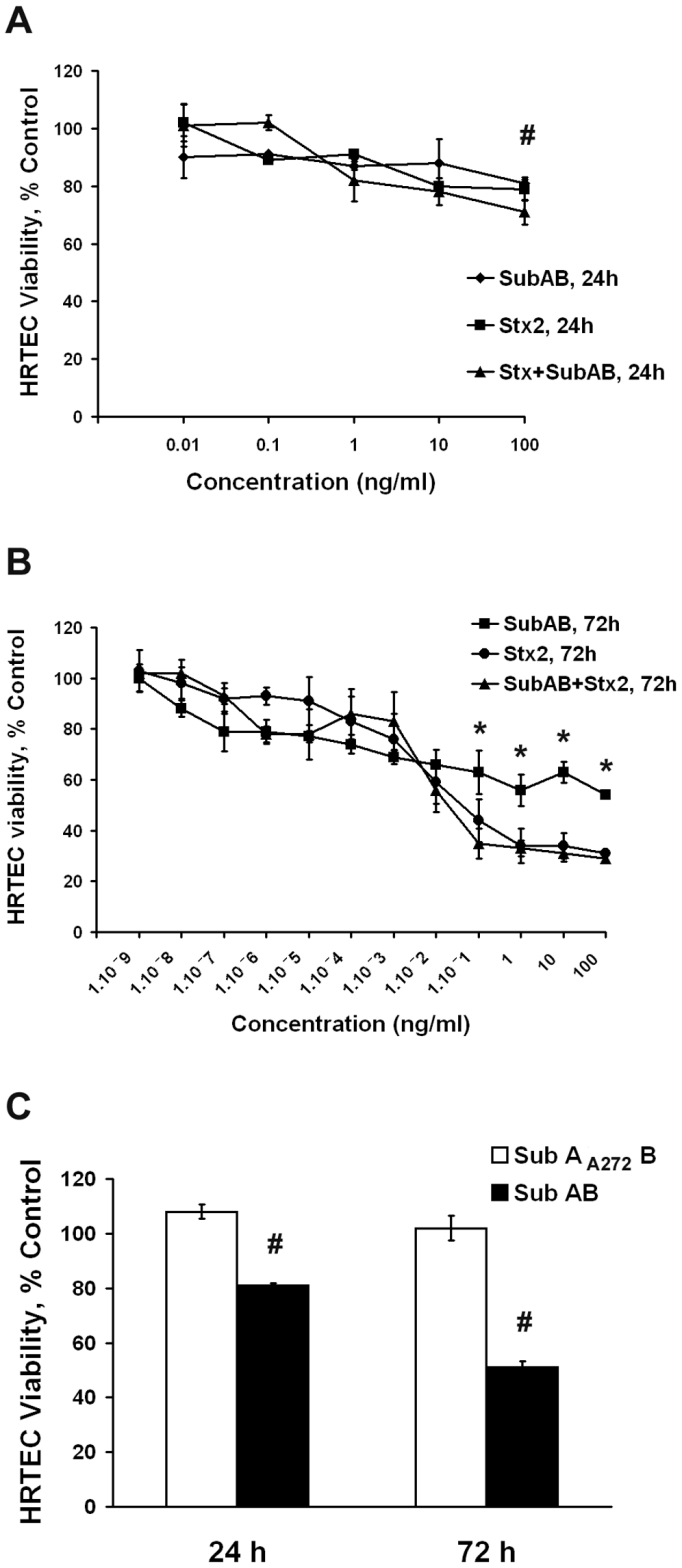
Effect of SubAB and Stx2 on cell viability by neutral red uptake assay. Both SubAB and Stx2 inhibited HRTEC viability in a dose dependent manner at 24(**A**) and 72 h (**B**). No potentiation of the cytotoxic effect was observed when cells were co-incubated with SubAB and Stx2, for 24 h (**A**). HRTEC viability was not significantly modified by the incubations with SubA_A272_B (100 ng/ml) for 24 or 72 h (**C**). Results are expressed as neutral red uptake percent, where 100% represents control cells without toxin treatment. Each point represents the mean ± SEM of three to five experiments. Student's *t*-tests indicate significant differences *p<0.05, for SubAB *vs* Stx2 treatments, and ^#^ *p*<0.05, for SubAB and/or Stx2 treated *vs* untreated control cells.

The cytotoxic activity of SubAB and Stx2 was evaluated on HRTEC by the measurement of cell viability using the neutral red uptake assay. For this purpose, confluent HRTEC were incubated with different dilutions of SubAB and Stx2, for 24 h and 72 h. Both SubAB and Stx2 inhibited cell viability in a time- and dose-dependent manner (**Figure 1**). Incubation with 100 ng/ml of Stx2 or SubAB for 24 h produced a significant reduction in cell viability (**Figure 1A**). No potentiation of the cytotoxic effect was observed when cells were co-incubated with different concentrations of both SubAB and Stx2 toxins at different times (**Figure 1A and 1B**). Incubation with 0.01 ng/ml of Stx2 for 72 h produced a 50% of inhibition of HRTEC viability, whereas SubAB caused an approximately 50% of inhibition in the concentration range of 1–100 ng/ml (**Figure 1B**). As shown in **Figure 1B**, Stx2 produced a significantly higher inhibition of cell viability than SubAB at concentrations equal to or greater than 0.01 ng/ml, while no significant differences were observed between the two toxins when HRTEC were exposed at doses less than or equal to 0.001 ng/ml. Incubation with the non-toxic mutant SubA_A272_B (100 ng/ml) for 24 or 72 h did not affect HRTEC viability (**Figure 1C**).

### Apoptosis

**Figure 2:**
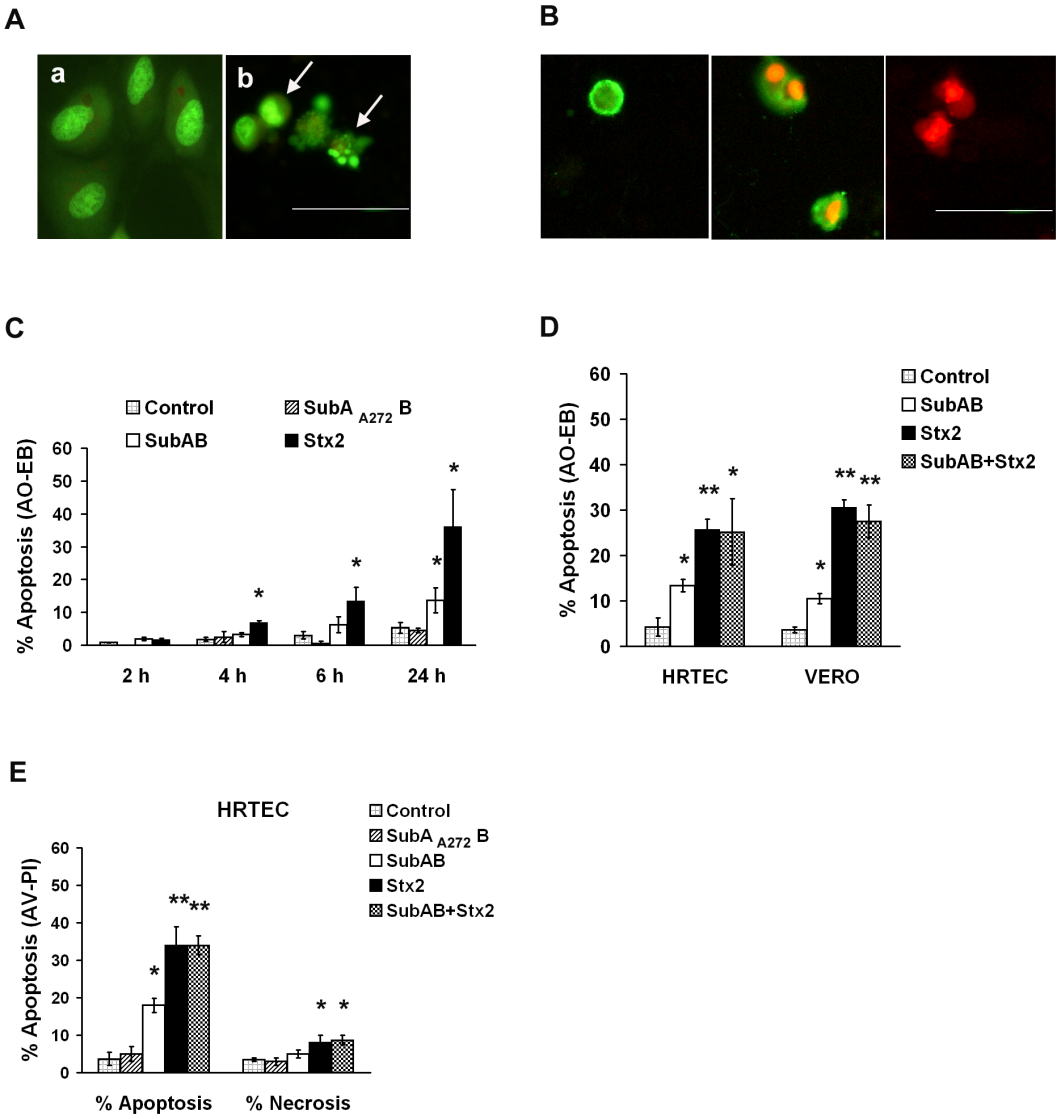
Percentage of apoptosis in HRTEC primary cultures and Vero cells exposed to SubAB and/or Stx2. Apoptosis was evaluated by acridine orange-ethidium bromide (AO-BE) staining (**A**, **C**, and **D**), and Annexin V-FITC - Propidium Iodide (AV-IP) (**B and E**) assays. Detection of apoptosis by AO/EB assay (**A**): live cells (**a**) show normal coloration of the nuclei, with green chromatin and organized structures; apoptotic cells present chromatin condensation and fragmentation (**b, white arrows**). Detection of apoptosis by AV-IP assay (**B**): early and late apoptotic cells were stained by AV (green) alone and by both AV and IP (green and red), respectively; while necrotic cells were stained by IP (red) alone. Bars in **A** and **B**: 50 µm. Exposure to SubAB or Stx2 (10 ng/ml each) induced apoptosis in HRTEC in a time dependent manner (**C**). Co-incubation with Stx2 and SubAB for 24 h did not potentiate the apoptosis and necrosis in HRTEC (**D, E**), nor in Vero (**D**) cultures. Each bar represents the mean ± SEM of three to five independent experiments. Student's *t*-tests indicate significant differences (*p<0.05 and **p<0.01) for SubAB and/or Stx2 treated *vs* untreated control cells.

To perform HRTEC proliferation rate and apoptosis assays at 24 h and earlier times, a dose of SubAB that killed about 50% of HRTEC in 72 h (10 ng/ml) was chosen. Apoptotic cells were detected by morphological appearance of the cells stained with AO-BE (**Figure 2A**) and by AV-PI staining (**Figure 2B**).

**Figure 2C** shows apoptosis assessed by AO-BE staining of HRTEC cultures exposed to SubAB or Stx2 (10 ng/ml, each), at different times. As well as effects on cell viability, both toxins stimulated the percentage of apoptotic cells in a time dependent manner, although Stx2 produced a significantly higher stimulation of apoptosis than SubAB on HRTEC primary cultures at equivalent doses (**Figure 2C**). A significant stimulation of the percentage of apoptotic cells was detected first after 4 h incubation with Stx2, and 24 h incubation with SubAB (**Figure 2C**). However, 24 h co-incubation with both toxins (**Figure 2D**) did not potentiate the apoptosis in HRTEC primary cultures nor in the Vero cell line. Moreover, higher stimulation of apoptosis was also observed in Vero cells exposed to Stx2, than in SubAB-treated cells (**Figure 2D**).

To analyze apoptosis and necrosis, the incorporation of AV-PI (**Figure 2E and 2B**) in HRTEC cultures exposed to SubAB and Stx2 was also examined, corroborating the results of apoptosis obtained using the AO-BE assay. As shown in **Figure 2E**, incubation with 10 ng/ml SubAB for 24 h significantly stimulated HRTEC apoptosis compared to control cells (% Apoptosis: 18±1.9 *vs.* 3.7±1.8, respectively, *p*<0.05). Furthermore, a higher percentage of apoptotic cells (34±5%) was observed in HRTEC exposed to the same dose of Stx2 (**Figure 2E**). Stx2 also produced a small but significant increase in the percentage of necrotic cells, while SubAB did not induce necrosis in HRTEC cultures (**Figure 2E**). Co-incubation with SubAB and Stx2 for 24 h did not potentiate their apoptotic or necrotic effects on HRTEC, similarly to the results shown for cell viability (**Figure 1A and 1B**) and apoptosis by AO-BE (**Figure 2D and 2E**).

In agreement with the results for cell viability above, incubation with the mutated SubA_A272_B did not stimulate HRTEC apoptosis compared to non-treated cells (**Figure 2C and 2E**).

### Cell proliferation rate

**Figure 3:**
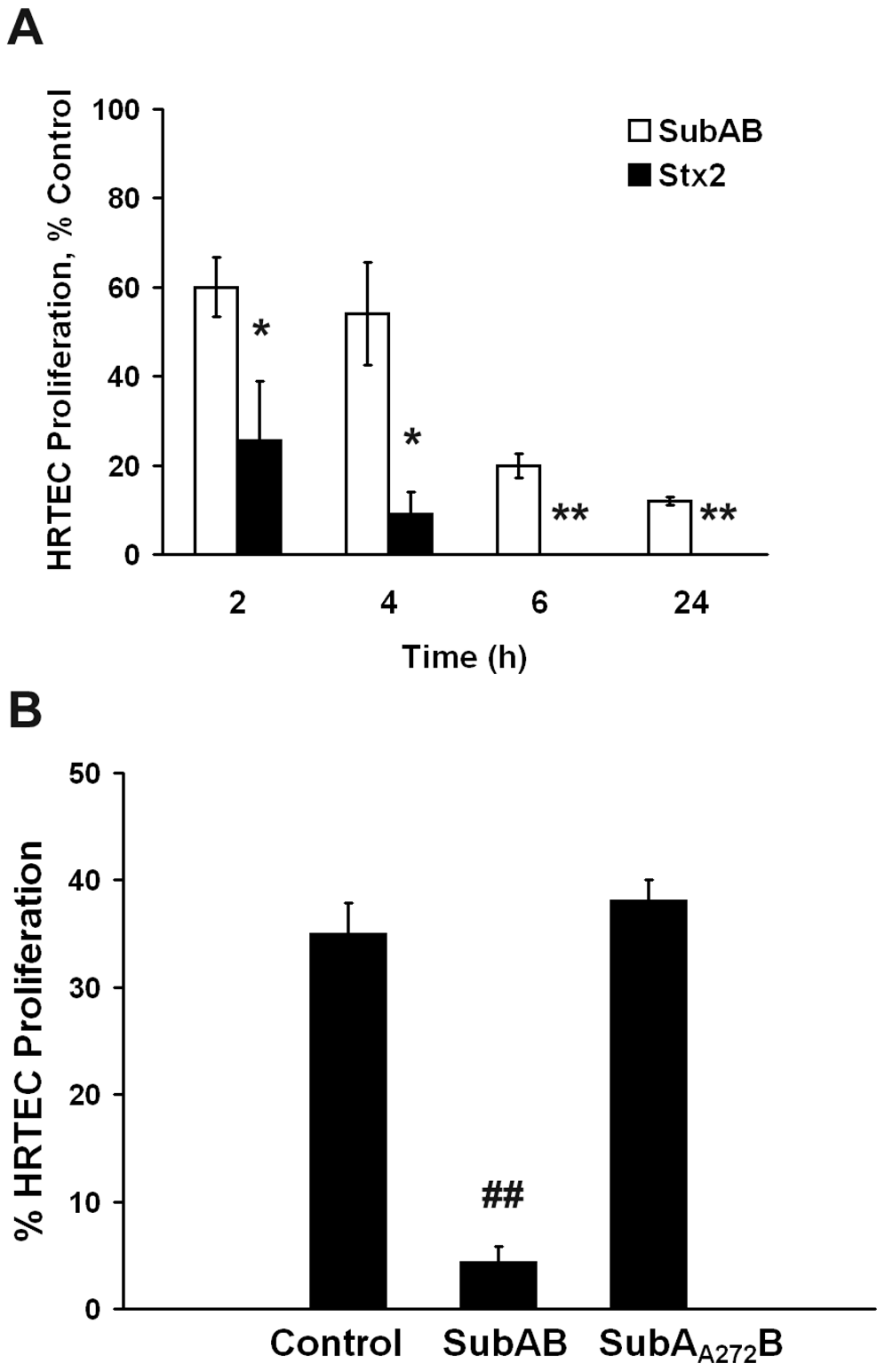
Cell proliferation rate measured by bromodeoxyuridine (BrdU) uptake in HRTEC primary cultures. HRTEC were incubated with 10/ml of either Stx2 or SubAB for different times (**A**). Both toxins produced a significant inhibition on the percentage of cell proliferation, compared to untreated control cells, in a time dependent manner, although HRTEC were more sensitive to Stx2 than SubAB. (**A**). Incubation with 10 ng/ml of SubA_A272_B did not modify BrdU uptake of HRTEC relative to untreated control cells (**B**). Each bar represents the mean ± SEM of three different experiments. Student's *t*-tests indicate significant differences (*p<0.05 and **p<0.01) for SubAB *vs* Stx2 treated cells and for SubAB or Stx2 treated *vs* untreated control cells (^##^ *p*<0.01).

Cell proliferation rate was measured by BrdU uptake in HRTEC, allowing determination of the DNA replication rate. Control HRTEC cultures grown in RPMI medium supplemented with endothelial cell growth supplement and 5% FBS, showed about 30 to 36% BrdU positive cells. As shown in **Figure 3A**, incubation with 10 ng/ml of either Stx2 or SubAB produced a significant inhibition of cell proliferation with respect to untreated control cells, in a time-dependent manner. However, Stx2 produced a significantly higher inhibition of cell proliferation compared to SubAB at all times studied. When HRTEC were incubated with Stx2 or SubAB for 2 h followed by pulse-labeling with BrdU for another 2 h, cell proliferation was reduced to about 25% and 60%, respectively, relative to untreated control cells. Moreover, cell proliferation was completely inhibited after 6 h exposure to Stx2, while residual proliferation remained after 24 h incubation with SubAB (**Figure 3A**). These results are consistent with those observed for cell viability and apoptosis, confirming that HRTEC were more sensitive to Stx2 than SubAB cytotoxic effects.

Incubation with 10 ng/ml of SubA_A272_B did not modify BrdU uptake by HRTEC cultures (**Figure 3B**).

## Discussion

In previous studies, SubAB toxin was found to be lethal for mice, resulting in extensive microvascular damage, thrombosis and necrosis in several organs, including the kidneys [6], [28], features observed in Stx-induced HUS in humans. The holotoxin was also proved to be highly toxic for several cell lines, including Vero cells [6], [23]–[27]. However, the role that SubAB plays in the pathogenesis of HUS remains to be elucidated. Production of SubAB or presence of the *subAB* genes has been detected in numerous STEC serotypes [7], [9], many of which have been associated with HUS cases around the world. All of these STEC strains lacked the locus of enterocyte effacement (i.e. they were LEE-negative) and, with the exception of one recent report [32] all strains produced SubAB as well as Stx1 and/or Stx2 [7], [9]. The role of Stx has been studied extensively in several animal models with the aim of demonstrating its role in the pathophysiology of HUS [15], [18], [19], [33]–[35]. Numerous studies in different cell types have also been performed to elucidate the mechanisms by which Stxs exert their effects on protein synthesis inhibition, and stimulation of cell apoptosis and necrosis [15], [16], [29], [36].

In the present work, we have studied the cytotoxic effects of SubAB on HRTEC, a primary culture of human renal proximal tubular epithelial cells. Treatments with SubAB were conducted in parallel with those performed with Stx2 under identical conditions, to compare the effects produced by the two toxins on HRTEC, and to investigate whether the effects of one toxin may influence the effects of the other. Here, we show for the first time that SubAB significantly inhibited cell viability and cell proliferation rate, as well as stimulating apoptosis in HRTEC cultures, demonstrating a significant sensitivity to SubAB cytotoxin. However, HRTEC cultures were significantly more sensitive to the cytotoxic effects of Stx2 than those induced by SubAB. Apoptosis assays were also performed in Vero cells, which showed results comparable to those obtained in the primary HRTEC cultures. Furthermore, about 20% of cells in HRTEC cultures remain alive after 72 h of Stx2 exposure indicating that a subpopulation of HRTEC is relatively toxin resistant. Similar results were reported in HK-2 cells [37].

Taking account of the fact that serine protease activity is central to the mechanism of action of SubAB [6], non-proteolytic mutant SubA_A272_B was used as a negative control. Incubation of HRTEC with SubA_A272_B showed similar results to those for untreated control cells, confirming the specific effects produced by SubAB.

Furthermore, it was previously demonstrated that pretreatment with C-9, a specific inhibitor of glucosylceramide synthase, inhibited the biosynthesis of Gb3 in HRTEC [20] and in rat kidneys *in vivo* [19], and neutralized the cytotoxic effects of Stx2 [19], [20]. These results showed that the sensitivity of HRTEC to Stx2 is dependent on the presence of the receptor Gb3 on their surface. In contrast, SubAB binds with a very high degree of specificity to cell surface glycans terminating in α2-3-linked Neu5Gc [22]. Although humans are genetically unable to produce the sialic acid Neu5Gc, it was demonstrated that Neu5Gc is taken up from the diet and assimilated into several human tissues, including the renal tubular epithelium [38]. Therefore, our results suggest the presence of Neu5Gc in HRTEC.

Interestingly, no synergism was observed when HRTEC were co-incubated with both Stx2 and SubAB in any of our studies. Experiments carried out with Stx2 alone produced similar cytotoxic effects to those in which cells were co-treated with both Stx2 and SubAB toxins. These results are consistent with those recently reported by Amaral et al. [39], where no evidence of synergy was observed in human renal microvascular endothelial cells treated with a combination of SubAB and Stx2.

Although HRTEC primary cultures were highly sensitive to SubAB and Stx2 toxins, differences between their cytotoxic activities were observed. To analyze these mechanisms, we studied necrosis and apoptosis of HRTEC exposed to Stx2 and SubAB. Both toxins caused significantly more apoptosis than necrosis. While Stx2 increased apoptosis in a time-dependent manner as early as 4 h after treatment, SubAB caused apoptosis only after 24 h of treatment. Faster effects on cell proliferation were also observed in HRTEC exposed to Stx2 than in cells treated with SubAB. Differences in intracellular trafficking may play a role in susceptibility to toxin-mediated cytotoxicity. It is known that efficient retrograde transport of the toxin is necessary for Stx cytotoxic effects [12], [40]. In most cell types, the induction of apoptosis requires transport of enzymatically active Stx to the endoplasmic reticulum (ER) and activation of the ribotoxic stress response or induction of the UPR [37], [41]–[43]. It was established that like Stx, SubAB is also trafficked from the cell surface via the Golgi to the ER via a retrograde pathway. However, SubAB uses a distinct route through the Golgi [44], and its internalization and trafficking is exclusively clathrin-dependent [45], whereas Stx can also exploit the lipid raft transport pathway. Once in the ER lumen, SubAB cleaves GRP78 and induces ER stress and the UPR [23], [24]. Therefore, both Stx and SubAB may induce apoptosis through prolonged activation of the ER stress response. This mechanistic overlap, together with the possibility that Stx2 quikly achieve apoptotic machinery in HRTEC, masking the action of SubAB, may explain the lack of synergy between the two toxins.

Both SubAB and Stx2 significantly inhibited HRTEC proliferation, measured as BrdU uptake into the DNA, although SubAB caused a lesser effect than Stx2. It has been reported that interference with the cell cycle, which results in inhibition of cell proliferation and activation of cell cycle checkpoints, is often associated with the initiation of apoptosis [46]. It has been demonstrated that incubation of human HCT116 colon cancer cells with Stx1 induced the arrest of cells in S phase, followed by programmed cell death [47]. Other studies showed that Stx1 activated the ATM/p53-dependent DNA damage signaling pathway and induced apoptosis [48]. SubAB toxin was also demonstrated to induce cell cycle arrest in G1 phase, possibly through down-regulation of cyclin D1 due to a combination of translational inhibition and proteasomal degradation [26]. Therefore, the differential capacity of Stx2 and SubAB to inhibit HRTEC proliferation may be related to the different ability to cause apoptosis. However, the precise cytotoxic mechanisms implemented by both SubAB and Stx2 toxins are still under study.

In conclusion, the present work shows that primary cultures of human renal tubular epithelial cells are sensitive to the cytotoxic effects of SubAB. The action of Stx2 is predominant on SubAB activity, indicating that SubAB mechanisms could be masked by Stx2 in HRTEC. Further studies will be necessary to understand the mechanisms triggering in human host cells in response to the combined action of SubAB cytotoxin and Stx2 produced by STEC.
